# Association between Subclinical Low Serum 25(OH)D in Donors and Fatty Liver Disease in Recipients after Living Donor Liver Transplantation

**DOI:** 10.1155/2018/4508085

**Published:** 2018-03-19

**Authors:** King-Wah Chiu, Toshiaki Nakano, Tsung-Hui Hu, Kuang-Den Chen, Li-Wen Hsu, Hock-Liew Eng, Yu-Fan Cheng, Shigeru Goto, Chao-Long Chen

**Affiliations:** ^1^Division of Hepato-Gastroenterology, Department of Internal Medicine, Kaohsiung Chang Gung Memorial Hospital, Kaohsiung, Taiwan; ^2^Chang Gung University, College of Medicine, Taoyuan, Taiwan; ^3^Liver Transplantation Centre, Kaohsiung Chang Gung Memorial Hospital, Kaohsiung, Taiwan; ^4^Graduate Institute of Clinical Medical Sciences, Chang Gung University, Taoyuan, Taiwan; ^5^Division of General Surgery, Department of Surgery, Kaohsiung Chang Gung Memorial Hospital and Chang Gung University College of Medicine, Kaohsiung, Taiwan; ^6^Department of Pathology, Kaohsiung Chang Gung Memorial Hospital, Kaohsiung, Taiwan; ^7^Department of Diagnostic Radiology, Kaohsiung Chang Gung Memorial Hospital, Kaohsiung, Taiwan; ^8^Fukuoka Institution of Occupational Health, Nobeoka, Japan; ^9^Department of Nursing, Josal International University, Togane, Japan

## Abstract

To explore subclinical fatty liver disease (FLD) in donors as a possible mechanism leading to FLD in recipients of living donor liver transplantation (LDLT), we extracted thirty donor-recipient pairs' serum DNA and explored the presence of CYP2R1 single nucleotide polymorphism (SNP) rs10741657 and vitamin D receptor (VDR) SNP rs2228530 A/G alleles using real-time polymerase chain reaction. We measured the serum 25(OH)D concentrations and investigated the CYP2R1 and VDR genotypes of the donors and recipients before and after LDLT for comparison with the histological findings from the donors on wedge biopsy, the recipients' removed native liver, and selective liver biopsy after LDLT. There was a significant difference in low serum 25(OH)D concentration between the donors and recipients before LDLT and in the recipients before versus after LDLT (13.90 ± 8.85 versus 47.9 ± 14.88 versus 11.82 ± 10.36, *P* < 0.001), and significant difference in FLD was detected on wedge biopsy from the donors and the native liver from the recipients as well as the native liver and follow-up biopsy from the recipients (*P* < 0.001). CYP2R1 and VDR genotype were predominant, both for the AG and for the GG alleles. For the donor VDR SNP rs2228570, low serum 25(OH)D was significantly different between genotypes AA and AG (*P* = 0.024) as well as between genotypes AA and AG plus GG (*P* = 0.042). Our data suggest that donors' VDR rs2228570 AA alleles may play a major role in low serum 25(OH)D regarding pathological FLD in recipients after LDLT.

## 1. Introduction

Nonalcoholic steatohepatitis is strongly associated with low serum 25(OH)D [1–3] and affects patients present with progressive hepatic fibrosis, particularly chronic hepatitis C infection [4, 5]. Therefore, hepatocytes appear to be a target that affects serum 25(OH)D levels via the VDR [6]. In the field of the liver transplantation, the new liver from the donor contributes its own characteristics such as CYP2C19 [7], CYP3A4, CYP3A5, MDR-1 [8], and IL28B [9] that can be detected in the peripheral blood of the recipients. If the donor presents with characteristics of fatty liver disease (FLD), the recipient will likely demonstrate fatty changes after living donor liver transplantation (LDLT). Because individuals with severe fatty liver or evidence of nonalcoholic steatohepatitis should not be donors for LDLT [10], here we aimed to explore the evidence of subclinical FLD in donors as a possible mechanism leading to recipients developing fatty liver after LDLT.

## 2. Materials and Methods

### 2.1. Study Population

Thirty adult donor-recipient pairs who underwent LDLT as part of our liver transplantation program were randomly enrolled in the study. Briefly, the mean patient age was 34.5 years (range, 23–59 years) in donors and 57.3 years (range, 35–69 years) in recipients. Subjects included 14 male and sixteen female donors and 21 male and nine female recipients. The causes of liver transplantation were hepatitis B virus- (HBV-) related liver disease in 25 cases (including four cases of end-stage liver disease [ESLD], 16 of hepatomas, and five of bleeding esophageal varices); primary biliary cirrhosis in three cases; one case of autoimmune hepatitis; and one case of cryptogenic liver cirrhosis.

The inclusion criteria were adult donor-recipient pairs for LDLT of whom the recipients underwent selective liver biopsy after LDLT. All of the donors and recipients provided written informed consent. The exclusion criteria were a history of diabetes mellitus or thyroid disorder; current or past excessive alcohol intake (defined as >30 g/day in males and >20 g/day in females); treatment with drugs affecting vitamin D; positive serum anti-hepatitis C virus; pediatric recipients that underwent LDLT; and having undergone deceased donor liver transplantation in either childhood or adulthood.

### 2.2. Clinical Assessments

Clinical profiles included the following: age, sex, body weight (kg), body height (cm), body mass index (BMI) (kg/m^2^), ideal body weight (kg), cholesterol (mg/dL), triglycerides (mg/dL), fasting glucose (mg/dL), alanine aminotransferase (ALT) (IU/L), and gamma-glutamyl transpeptidase (IU/L) of the donors and recipients before LDLT (Table 1). Imaging evaluations included ultrasonography (findings of which were defined as negative, mild, moderate, or severe degree of fatty liver) and computed tomography and magnetic resonance imaging (findings of which were defined as negative, <5%, 5–10%, and >10% fatty liver) for the potential donors before LDLT and at 1- and 6-month follow-up in the recipients after LDLT.

### 2.3. Laboratory Assessment

#### 2.3.1. Serum 25(OH)D

Serum 25(OH)D levels were investigated in the donors and recipients before LDLT and in the recipients 1 and 3 months after. The serum 25(OH)D was measured using a 25(OH) vitamin D Enzyme-Linked Immunosorbent Assay kit (ENZO, Enzo Life Sciences Inc., NY, USA). The optical density of each well at 405 nm was determined within 30 min using a microplate reader. Each sample was assayed in duplicate by a single operator to assess interassay precision. All of the data were processed by an immunoassay software package utilizing a four-parameter logistic curve fitting program. By definition, serum 25(OH)D levels ≥ 30 ng/mL (75 nmol/L) were considered within normal limits in our study.

#### 2.3.2. VDR and CYP2R1 Polymorphisms

Genomic DNA was extracted from the peripheral blood mononuclear cells of the donors and recipients before LDLT and in the recipients 1–3 months after LDLT using a QIAamp DNA Blood Mini Kit (Qiagen, Hilden, Germany). Genotyping was performed to detect the single nucleotide polymorphisms (SNPs) VDR rs2228570 and CYP2R1 rs10741657 using a ready-to-use, manufacturer-validated, predesigned allele discriminating TaqMan SNP assay for polymerase chain reaction (PCR) amplification reactions in clear optical 96-well plates on a 7500 Fast Real-Time PCR system (Applied Biosystems International, Foster City, CA, USA) according to the manufacturer's instructions. The SNPs were selected according to A/G allele frequencies and functional clinical implications. All genotypes of the VDR SNP rs2228570 and CYP2R1 SNP rs10741657 were assayed in duplicate to assess interassay precision.

### 2.4. Ethics

Written informed consent was obtained from each participant. The study protocol conformed to the ethical guidelines of the Declaration of Helsinki and was approved by the ethics review committee of Chang Gung Memorial Hospital (number 201600292B0). None of the transplant donors or recipients was from a vulnerable population.

### 2.5. Statistics

Data were analyzed using the Statistical Package for the Social Sciences (v. 22.0 for Windows; IBM Corp., Armonk, NY, USA). Student's *t*-test was used to compare the clinical parameters of the donors and recipients before and after the LDLT. Fisher's exact test was used to compare the histological diagnosis between wedge biopsy of the donors and follow-up liver biopsy of the recipients after LDLT. The SNP allele differences in the VDR rs2228570 and CYP2R1 rs10741657 genotypes of the recipients and donors were performed at different time points using the McNemar–Bowker test. Differences were considered statistically significant at values of *P* < 0.05.

## 3. Results

### 3.1. Subclinical Low Serum 25(OH)D

**Figure 1:**
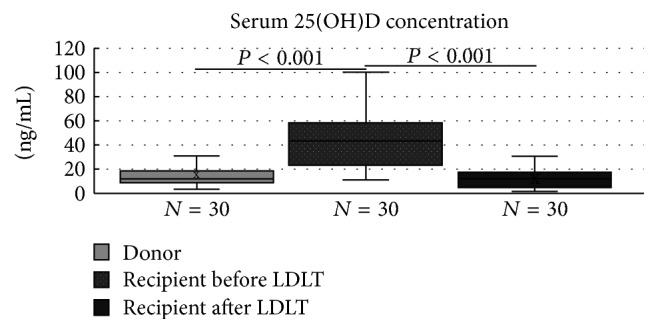
Difference in serum 25(OH)D levels of donor versus recipient before living donation liver transplantation (LDLT) and of recipient before versus after LDLT (*P* < 0.001).

There was a significant difference in the low serum 25(OH)D concentrations between the donors and recipients before LDLT (13.90 ± 8.85 versus 47.9 ± 14.88; *P* < 0.001) and in the recipients before versus after LDLT (47.9 ± 14.88 versus 11.82 ± 10.36; *P* < 0.001) (Figure 1). None of the clinical parameters, including BMI, ideal body weight, and indexes for metabolic disorders (cholesterol, triglyceride, and fasting glucose), differed statistically between the donors and recipients (Table 1).

### 3.2. Histology Plus Imaging Documented FLD

In the donor group, there was a 60% (18/30) fatty liver disease diagnosis by wedge biopsy (46.7%, 14/30) and by imaging (40%, 12/30) with eight cases (26.7%) overlapping. The native liver was resected and only 3.3% (1/30) of cases were associated with 10% fatty liver in the recipient. In the recipients after LDLT, there was a 63.3% (19/30) rate of fatty liver diagnosis by follow-up selective liver biopsy (40%, 12/30) and by imaging (40%, 12/30) with five cases (16.7%) overlapping. On the liver tissue histology and imaging studies, FLD differed significantly between the wedge biopsy in the donors and the native liver of the recipients (*P* < 0.001) as well as the native liver and the follow-up selective biopsy of the recipients (*P* < 0.001) (Table 2). By the wedge biopsy proven FLD in liver donors, there was statistical difference in terms of serum 25(OH)D levels between liver donors who have FLD (10.76 ± 4.97, *n* = 14) and subjects without (16.64 ± 7.62, *n* = 16) (*P* = 0.0078) (Table 3).

### 3.3. Comparison with CYP2R1 rs10741657/VRD rs2228530 and Serum 25(OH)D

**Figure 2:**
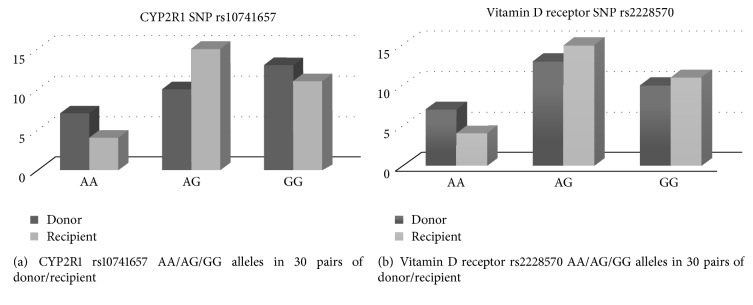


Regarding CYP2R1 SNP rs10741657, the donors and recipients predominantly had AG alleles (14/13) and GG alleles (12/11) but sporadically had AA alleles (4/6) (Figure 2(a)). Of the VDR SNP rs2228530 in the donors and recipients, a similar distribution was seen with predominance of AG alleles (12/15) and GG alleles (10/11) but sporadic AA alleles (7/4) (Figure 2(b)). For the donors, the VDR SNP rs2228570 AA genotype was explored with low serum 25(OH)D concentration compared to those of the AG genotype (*P* = 0.024); and the low serum 25(OH)D showed also significant difference between the genotype AA and genotype AG plus GG (*P* = 0.042) (Table 4). However, there was no significant distribution by genotype of CYP2R1 rs10741657 in the donors or recipients after LDLT.

## 4. Discussion

By definition, “subclinical” indicates the presence of a disease without the manifestation of symptoms and may be an early stage in the evolution of a disease. The current results suggest that the donors were doing well without evidence of signs of metabolic syndrome such as a high BMI or elevated cholesterol, triglyceride, or fasting glucose levels. In our study, the mean serum 25(OH)D concentration was <25 ng/mL in the healthy donors, representing a subclinical low serum 25(OH)D. A recent report suggested that a low serum 25(OH)D may be highly associated with nonalcoholic fatty liver disease or metabolic syndrome recognization [2], in which situations he/she should not be a liver donation followed by the donor evaluation in the LDLT setting. Too many studies to explore the nonalcoholic fatty liver disease or nonalcoholic steatohepatitis might be progressed to hepatic fibrosis and liver cirrhosis [11]. The complicating outcomes were more severe and mortality rates were higher in cases of nonalcoholic steatohepatitis particularly in cases of chronic hepatitis C viral infection [12]. To explore the role of 25(OH)D in the mechanism of FLD, we excluded the evidence of chronic C hepatitis relative to end-stage liver disease and alcoholic liver cirrhosis associated with metabolic disorder in our study. A report of the serum 25(OH)D concentration changing significantly from within the normal limit to a low serum level after LDLT would be the first in the literature. For the FLD investigation of donor livers, imaging studies including computed tomography and magnetic resolution imaging showed that >15% fatty liver required a liver biopsy before transplantation and >20% fatty component to be withdraw for liver donation [13, 14]. In our study, 60% (18/30) of cases were associated with FLD, and most had a <5% to a 5–10% fatty component. We used the term “subclinical low serum 25(OH)D with mild degree FLD” to identify such potential donors in this study. According to our previous studies, the characteristic stem cells of liver grafts may carry the specific signal that is transmitted to the recipients [15, 16]. Barchetta et al. reported that VDR and CYP2R1 may be closely associated with low serum 25(OH)D, particularly in cases of nonalcoholic steatohepatitis [6]. On the outcome of LT patients, 19 (63.3%) recipients presenting with mild degree abnormality of serum alanine aminotransferase around 48 to 78 IU/L during clinical followed up. Only 4 (13.3%) recipients experience mild rejection controlled by appropriate immunosuppressive agents, and apparently serum 25(OH)D has no significant effect on organ rejection. In our study, VDR SNP rs2228570 in the donors may carry a signal of FLD present with a low serum 25(OH)D, particularly in cases of AA alleles. This is also a new finding in the current study. On the other hand, CYP2R1 rs10741657 could not have a significant interpretation in our study. Of course, none of our patients met the criteria for nonalcoholic steatohepatitis. Several studies have showed that decreased vitamin D in patients with HBV, primary biliary cholangitis, and autoimmune hepatitis [17–19], but all of the underlying diseases should be corrected after liver transplantation. The current study showed that the evidence of the subclinical FLD from the donor may be one of the major factors to provide the possibility of the FLD to the recipients after LDLT.

**Figure 3:**
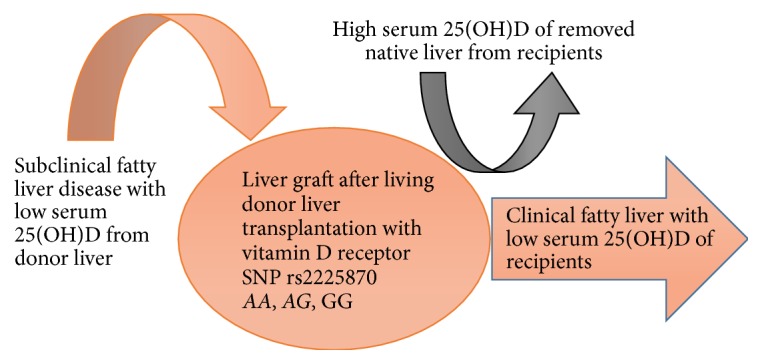
Graphic conclusion: a subclinical low serum 25(OH)D level is associated with VDR rs2228570, particularly the AA genotype, of the donors, which may play a major role in FLD in recipients after LDLT.

Our study has some limitations. The case collection may need to be enhanced over time. The other important factor CYP27A1 SNP is under ongoing study to be present in the future. We try to summarize a graphic conclusion in Figure 3. In conclusion, a subclinical low serum 25(OH)D level is associated with VDR rs2228570, particularly the AA genotype, of the donors, which may play a major role in FLD in recipients after LDLT.

## Figures and Tables

**Table 1:** Clinical profiles of donors and recipients before living donor liver transplantation.

Category	Donors (*n* = 30)	Recipients (*n* = 30)	*P* value
Gender (male/female)	14/16	21/9	0.115^*∗*^
Age (years)	34.47 ± 12.73	57.3 ± 12.81	0.982
Body height (cm)	165.54 ± 9.26	164.34 ± 2.33	0.715
Male	170.70 ± 1.56	167.27 ± 6.35	0.725
Female	161.03 ± 5.52	157.50 ± 6.93	0.173
Body weight (kg)	62.26 ± 3.96	63.63 ± 12.73	0.005
Male	65.69 ± 8.06	53.67 ± 15.88	0.014
Female	59.26 ± 5.37	63.63 ± 12.73	0.243
Body mass index	22.77 ± 4.00	23.32 ± 4.67	0.540
Male	22.67 ± 3.14	24.17 ± 3.07	0.730
Female	22.85 ± 3.59	21.32 ± 4.46	0.691
Idea body weight	59.67 ± 9.48	59.67 ± 3.89	0.632
Male	65.69 ± 1.20	63.11 ± 4.74	0.712
Female	54.41 ± 3.68	51.64 ± 4.59	0.736
Cholesterol, total (mg/dL)	159.03 ± 6.36	146.03 ± 74.95	0.080
Triglycerides (mg/dL)	95.63 ± 33.94	117.98 ± 18.38	0.411
Glucose (FG) (mg/dL)	91.3 ± 3.54	105.90 ± 20.51	0.157
ALT (IU/L)	19.00 ± 6.36	65.30 ± 17.68	0.000
GGT (IU/L)	14.70 ± 0.71	49.30 ± 13.44	0.000
Parathyroid hormone level	38.31 ± 9.81	40.09 ± 10.65	0.721
Liver biopsy			
Sample season^*∗∗*^	7 : 8 : 8 : 7	6 : 7 : 8 : 9	
Presence of inflammation (+/−)	1/19	4/16^*∗∗∗*^	0.094

^*∗*^Fisher's exact test (two-sided). Group statistics by independent samples *t*-test. ALT, alanine aminotransferase; FG, fasting glucose; GGT, gamma-glutamyl transpeptidase. ^*∗∗*^Spring : Summer : Autumn : Winter; ^*∗∗∗*^4 cases presenting with inflammation in liver biopsy were related to the mild degree rejection.

**Table 2:** Fatty liver disease diagnosis made intraoperatively by graft wedge biopsy, imaging on preoperative evaluation of donors, and by follow-up selective liver biopsy and imaging study on the recipients after living donor liver transplantation.

Fatty liver disease	Donors, *n* = 30 (%)		Recipients, *n* = 30 (%)		
	Wedge bx	Imaging	Native	F-U bx	Imaging
<5%	10 (33.3)	8 (26.7)	0 (0)	8 (26.7)	6 (20.0)
5–10%	2 (6.7)	3 (10)	1 (3.3)	3 (10)	5 (16.7)
>10%	2 (6.7)	1 (3.3)	0 (0)	1 (3.3)	1 (3.3)
Total	14 (46.7)	12 (40)	1 (3.3)	12 (40)	12 (40.0)

Bx + Imaging	18 (60)^a^		1 (3.3)^a,b^	19 (63.3)^b^	

^a^ *P* < 0.001; ^b^*P* < 0.001, Fisher's exact test (two-sided); Bx, liver biopsy; F-U, follow-up; image Dx, imaging diagnostic methods including ultrasonography, computed tomography, and magnetic resonance imaging.

**Table 3:** Serum 25(OH)D level between 30 liver donors by wedge biopsy proven with fatty liver disease (FLD) and subjects without.

	FLD (+)	FLD (−)	*P* value
	*n* = 14	*n* = 16	
Serum 25(OH)D	10.76 ± 4.97	16.64 ± 7.62	0.0078

Student's *t*-test analysis.

**Table 4:** Comparison of serum 25(OH)D concentration and single nucleotide polymorphisms (SNP) of vitamin D receptor (VDR) and cytochrome P450 2R1 (CYP2R1) between the donors and recipients before living donor liver transplantation.

Single nucleotide polymorphism	Allele	*N* (%)	25(OH)D ng/mL
*Donor*			
VDR rs2228570	AA	7 (23.3)	10.60 ± 3.67^a,b^
	AG	13 (43.3)	16.07 ± 8.14^a,b^
	GG	10 (33.3)	13.38 ± 6.95^b^
CYP2R1 rs10741657	AA	4 (13.3)	9.23 ± 6.93
	AG	15 (50.0)	15.08 ± 7.79
	GG	11 (36.7)	13.98 ± 5.92
*Recipient*			
VDR rs2228570	AA	4 (13.3)	79.31 ± 45.41
	AG	15 (50.0)	39.71 ± 22.89
	GG	11 (36.7)	47.63 ± 30.10
CYP2R1 rs10741657	AA	7 (23.3)	52.25 ± 16.66
	AG	10 (33.3)	43.22 ± 30.54
	GG	13 (43.3)	49.15 ± 37.75

^a^AA versus AG, *P* = 0.024; ^b^AA versus AG + GG, *P* = 0.042, Fisher's exact test.

## Abbreviations

ALT: Alanine aminotransferase
BMI: Body mass index
CYP2R1: Cytochrome P450 subfamily IIR1
ESLD: End-stage liver disease
FG: Fasting glucose
FLD: Fatty liver disease
GGT: Gamma-glutamyl transpeptidase
HBV: Hepatitis B virus
LDLT: Living donor liver transplantation
PCR: Polymerase chain reaction
SNPs: Single nucleotide polymorphisms
25(OH)D: 25-Hydroxyvitamin D
VDR: Vitamin D receptor.
